# Analysis of 470,000 exome-sequenced cases and controls fails to identify any genes impacting risk of developing affective disorder

**DOI:** 10.1017/neu.2025.10025

**Published:** 2025-06-30

**Authors:** David Curtis

**Affiliations:** UCL Genetics Institute, UCL, Darwin Building, Gower Street, London, WC1E 6BT, UK

**Keywords:** Depression, affective disorder, exome, rarevariant, gene

## Abstract

**Objective:**

A previous analysis of 200,000 exome-sequenced UK Biobank participants using weighted burden analysis of rare, damaging variants failed to identify any genes associated with risk of affective disorder requiring specialist treatment. Exome-sequence data has now been made available for the remaining 270,000 participants and a two-stage process was applied in order to test for association in this second sample using only genes showing suggestive evidence for association in the first sample.

**Methods:**

Cases were defined as participants who reported having seen a psychiatrist for ‘nerves, anxiety, tension or depression’. Exhaustive testing of the first sample was carried out using rare variant analyses informed by 45 different predictors of impact of nonsynonymous variants. The 100 genes showing the strongest evidence for association were then analysed in the second sample using the same predictor as had been most statistically significant in the first sample.

**Results:**

The results for the 100 nominated genes conformed closely with the null hypothesis, with none approaching statistical significance after correction for multiple testing.

**Conclusion:**

Risk of common affective disorder, even if severe enough to warrant specialist referral, is not sufficiently impacted by effects of rare variants in a small enough number of genes that effects can be detected even with large sample sizes. Actionable results might be obtained with a more extreme phenotype but very significant resources would be required to achieve adequate power. This research has been conducted using the UK Biobank Resource.

Significant outcomes

- In spite of a very large sample, the study fails to implicate any specific genes in the aetiology of affective disorder.
- The results raise questions about the optimal study design for identifying genetic factors impacting affective disorder.

Limitations

- The phenotype used consisted of self-report of being referred to a psychiatrist.
- The phenotype was not diagnosis-specific, though shown to be genetically related to the diagnosis of depression used in other large scale studies.

## Introduction

Studies of large exome-sequenced case-control cohorts have been successful in identifying genes harbouring extremely rare variants with large effects on the risk of schizophrenia and these results may plausibly assist the development of novel treatments (Palmer *et al*., 2022; Curtis, 2022a; Liu *et al*., 2023; Heinzer & Curtis, 2024; Singh & The Schizophrenia Exome Meta-Analysis (SCHEMA) Consortium, 2022). However when a similar study design was applied to a phenotype related to affective disorder in a sample of 200,000 exome-sequenced UK Biobank participants (https://www.ukbiobank.ac.uk/about-biobank-uk/), including nearly 23,000 cases, no gene approached statistical significance after correction for multiple testing and the results were reported to conform closely to those expected under the null hypothesis (Curtis, 2021). The phenotype in question was defined as answering positively to the question ‘Have you ever seen a psychiatrist for nerves, anxiety, tension or depression?’. Given that UK Biobank participants consist of volunteers who tend to be relatively healthy and middle-aged or elderly, with under-representation of schizophrenia, personality disorder, substance misuse and learning disability, it was argued that the bulk of those answering positively to this question would have a mood disorder. It was also argued that, because in Britain most cases of anxiety and/or depression are treated in primary care without referral to a psychiatrist, people responding positively would have had relatively severe illness. Another reason for choosing this item as the phenotype of interest was because the question was answered by almost all participants, whereas other psychiatric phenotypes were only available for smaller numbers.

The fact that a similar sample size was sufficient to identify genes implicated in schizophrenia pathogenesis but not this mood disorder phenotype could be explained by schizophrenia having a higher heritability than depression, and also possibly with major effects concentrated in a smaller number of genes. This mirrors the findings of genome-wide association studies using common variants, for which depression has a lower yield of statistically significant findings than schizophrenia (Kendall *et al*., 2021).

Exome sequence data for the remaining 270,000 UK Biobank participants has now been released, meaning that a two-stage strategy could be applied. Even though analysis of the first 200,000 did not yield any genes producing results significant after correcting for multiple testing of 20,000 genes, a number of genes did produce fairly small p values of 0.001 or less. Although at least some of these would be chance findings it might be that some of these smaller p values could reflect a true effect. One might select those genes which produced the lowest p values in the first sample and test only these genes in the second sample, which would then require a much less rigorous correction for multiple testing than having to correct for all 20,000 genes. This strategy has been successfully applied to three other common, clinically important phenotypes – hypertension, hypercholesterolaemia and type 2 diabetes (Curtis, 2023a, b, 2024). For each of these, only the few dozen genes significant at *p* < 0.001 in the 200,000 sample were tested for association in the 270,000 sample. For each phenotype, this allowed the identification of a small number of genes which demonstrated clear evidence of association which was statistically significant after correction for the number of genes tested.

This two-stage strategy was further modified before being applied to the affective disorder phenotype. Previously, in order to test the strength of evidence for association of each gene a weighted burden analysis had been used. To implement this, a system of weights is applied to each variant depending on the category of predicted effect by Variant Effect Predictor (VEP), such as loss of function (LOF), nonsynonymous, synonymous, intronic, etc (McLaren *et al*., 2016). For nonsynonymous variants the weight is further adjusted using the predicted impact of the amino acid change according to SIFT and PolyPhen (Kumar *et al*., 2009; Adzhubei *et al*., 2013). A weight is also devised according to the rarity of the variant and then the functional weight and rarity weight are multiplied together. For each gene, each subject is assigned a weighted burden score which consists of the sums of the weights of the variants which they carry and logistic regression is used to see if this score is associated with the phenotype. However, the weighting scheme is devised in advance and an obvious problem is that if the assigned weights do not adequately reflect the actual biological impacts of the different variant types then power will be reduced. A separate study has shown that that there is variability across genes and phenotypes as to the relative contributions of LOF and nonsynonymous variants, and also for the different predictors of impact of nonsynonymous variants (Curtis, 2022b). Thus, for some genes predictors such as SIFT and PolyPhen might work fairly well while for other genes a different prediction method might be superior. In order to address this issue, it was decided to reanalyse the 200,000 sample using repeated analyses with different prediction methods. For each gene, the weights for LOF and nonsynonymous variants would be fitted separately and multiple analyses would be performed using different predictors of impact of nonsynonymous variants. Then the genes producing the most highly significant results overall would be carried forward to be analysed in the second sample, using for each gene only the predictor which had yielded the most significant result.

It was decided to enter 100 gene-predictor pairs into the second stage of the analysis. The expectation was that if variants in a gene were actually associated with the phenotype then the best-performing predictor for that gene might produce evidence for association significant at *p* < 0.0005 or so in the first stage. Subsequently in the second stage a result would only need to produce a *p* value of 0.05/100 = 0.0005 or less in order to be regarded as statistically significant after correction for multiple testing. There would be an expectation that through the winner’s curse effect there might be a weaker association evident in the second sample, although this might be somewhat mitigated by the fact that the second sample is larger than the first.

It was hoped that applying this extensive search to find suggestive evidence for association in the first sample followed by attempts at confirmation in the second sample could lead to the identification of genes involved in susceptibility to affective disorder.

## Materials and methods

Relevant UK Biobank phenotype fields had been downloaded along with the variant call files for 200,632 subjects who had undergone exome-sequencing and genotyping by the UK Biobank Exome Sequencing Consortium using the GRCh38 assembly with coverage 20X at 95.6% of sites on average (Szustakowski *et al*., 2021). The UK Biobank Research Analysis Platform was used to access the Final Release Population level exome variants in PLINK format for 469,818 exomes which had been produced at the Regeneron Genetics Center based on DNA extracted from stored blood samples and using the protocols described here: https://dnanexus.gitbook.io/uk-biobank-rap/science-corner/whole-exome-sequencing-oqfe-protocol/protocol-for-processing-ukb-whole-exome-sequencing-data-sets (Backman *et al*., 2021). All variants were then annotated using the standard software packages VEP, PolyPhen and SIFT (Kumar *et al*., 2009; Adzhubei *et al*., 2013; McLaren *et al*., 2016). To obtain population principal components reflecting ancestry, version 2.0 of *plink* (https://www.cog-genomics.org/plink/2.0/) was run with the options – *maf 0.1 – pca 20 approx* (Chang *et al*., 2015; Galinsky *et al*., 2016). UK Biobank had obtained ethics approval from the North West Multi-centre Research Ethics Committee which covers the UK (approval number: 11/NW/0382) and had obtained informed consent from all participants. The UK Biobank approved an application for use of the data (ID 51119) and ethics approval for the analyses was obtained from the UCL Research Ethics Committee (11527/003).

The phenotype was determined according to how participants had responded in their initial assessment to the touchscreen question: ‘Have you ever seen a psychiatrist for nerves, anxiety, tension or depression?’ Those answering ‘Yes’ were taken to be cases and all those answering ‘No’ were taken to be controls. No attempt was made to screen out controls who might have had some other psychiatric diagnosis.

In order to gain further insight into the appropriateness of this phenotype, its genetic correlation with major depressive disorder (MDD) was determined. Firstly, summary statistics for association between the phenotype and the UK Biobank Axiom array single nucleotide polymorphisms were calculated for 415,318 participants reporting White British ancestry (Bycroft *et al*., 2018). Next, the Psychiatric Genetic Consortium site (https://pgc.unc.edu/for-researchers/download-results/) was accessed to obtain summary statistics from a genome wide association study (GWAS) of MDD using 135,458 cases and 344,901 controls, of whom 14,260 cases and 15,480 controls were UK Biobank participants (Wray *et al*., 2018). Finally, the genetic correlation between the two sets of summary statistics was calculated using linkage disequilibrium score analysis implemented in the LDSC programme (Bulik-Sullivan *et al*., 2015a, b).

SCOREASSOC was used to carry out logistic regression analysis to test whether, in each RefSeq gene, sequence variants which were rarer and/or predicted to have more severe functional effects occurred more commonly in cases than controls (Curtis, 2012). Attention was restricted to rare variants with minor allele frequency (MAF) ≤ 0.01. All genes having at least one such variant were tested, consisting of 22,560 genes in total. For each gene, two scores were produced for each subject, a LOF variant score and a nonsynonymous variant score. For every LOF variant and nonsynonymous variant, a weight based on MAF was assigned using the previously described method to fit a parabolic function such that variants with MAF = 0.01 were given a weight of 1 while very rare variants with MAF close to zero were given a weight of 10 (Curtis, 2012). For each subject, the LOF variant score consisted of the sum of these weights for any LOF variants which that subject carried, consisting of stop, frameshift and essential splice site variants. For every nonsynonymous variant, additional functional weights based on predicted impact were assigned using different methods intended to predict the likely pathogenicity of a variant. These functional weights consisted of the rank scores for each of 43 different prediction and conservation methods as provided for all possible nonsynonymous variants in dbNSFP v4 (Liu *et al*., 2020). Two additional functional weights were obtained for annotations using AlphaMissense by running VEP with the options *b – canonical –regulatory – plugin AlphaMissense* (Cheng *et al*., 2023). This outputs two AlphaMissense annotations, a raw score and a categorisation of likely pathogenic, likely benign or ambiguous, these three categories being converted to numerical scores of 2, 0 or 1 respectively. Thus, each nonsynonymous variant had a total of 45 different functional weights, produced by 45 different prediction methods. These were multiplied by the weight due to MAF to give 45 different overall weights for that variant. The nonsynonymous variant score for the subject would consist of the sum of the weights for all nonsynonymous variants that subject carried and there would be 45 different versions of this score, depending on which annotation method was used.

For variants on the X chromosome, hemizygous males were treated as homozygotes. Variants were excluded if there were more than 10% of genotypes missing or if the heterozygote count was smaller than both homozygote counts. If a subject was not genotyped for a variant then they were assigned the subject-wise average score for that variant.

The first phase of analysis was applied to the initial cohort of 200,000 exome-sequenced participants. For each gene, a logistic regression analysis was carried out to test whether the gene-wise LOF score and/or nonsynonymous score were associated with phenotype. To do this, the log likelihood was calculated for the null hypothesis model using only sex and the first 20 population principal components to predict phenotype status. Then the log likelihood was calculated for the alternative hypothesis model additionally including the LOF score and nonsynonymous score. Twice the difference between these two log likelihoods was taken to be a likelihood ratio statistic expected to be distributed as chi-squared with two degrees of freedom. The p value obtained for this statistic was converted to a minus log10 P (MLP) value for convenience. For each gene, this process was repeated 45 times, for each of the different predictors of nonsynonymous variant pathogenicity. Then for each gene the predictor producing the highest MLP, termed MaxMLP, was identified.

Once a MaxMLP was obtained for every gene, the top 100 MaxMLPs were used to select gene-predictor pairs for the second phase of the analysis, to be carried out in the second sample of 270,000 participants. The same likelihood ratio test based on logistic regression was applied to see if the LOF and/or nonsynonymous score were associated with phenotype, but for each of the 100 genes only a single nonsynonymous score was used, using the predictor which had produced the highest MLP in the first sample. This meant that a total of only 100 tests for association would be performed.

## Results

The genetic correlation between the phenotype based on answering positively to the question about having seen a psychiatrist for ‘nerves, anxiety, tension or depression’ and the phenotype of MDD as used in the previous GWAS was calculated by LDSC as rg = 0.87.

For the first phase of analysis, there were 22,886 cases and 176,486 controls. 22,560 genes were analysed as described above and the 100 highest MaxMLPs ranged from 3.50 for *AKNAD1* to 6.03 for *AZIN1*. Results for all genes and all 45 tests are provided in Supplementary Table 1.

**Figure 1.**
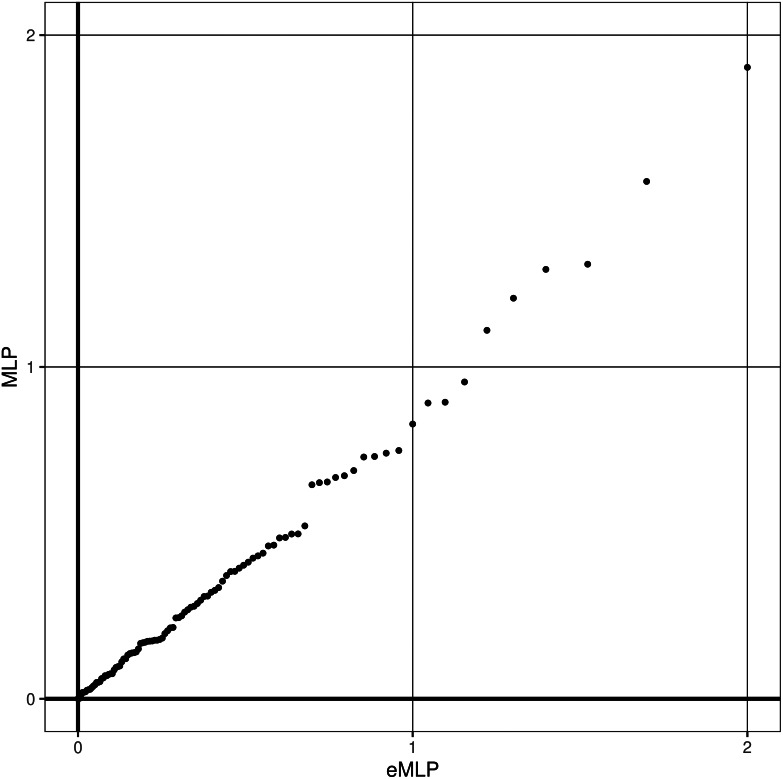
QQ plot of minus log10 Ps (MLPs) for rare variant analyses in 270,000 UK Biobank participants of 100 genes tested for association with referral for psychiatric treatment, showing observed against expected MLP for each gene. The null hypothesis expectation is that the results will fall on the *x* = *y* diagonal.

For the second phase of analysis there were 30,864 cases and 236,674 controls. The 100 genes nominated in the first phase were analysed to see if the LOF score and/or nonsynonymous score were associated with phenotypes, the nonsynonymous score being generated using the predictor which had produced the MaxMLP for each gene in question. The results obtained from this second phase are shown in Supplementary Table 2. A QQ plot of the MLPs obtained from these 100 analyses against the expected null hypothesis distribution is shown in Fig. 1. It can be seen that the results conform very closely to what would be expected under the null hypothesis that there are no genes for which the LOF score or nonsynonymous score is associated with the phenotype. The highest MLP produced by any gene is 1.90 for *MGAM*, equivalent to *p* = 0.012. In order for any result to be regarded as statistically significant, given that 100 genes were tested, the MLP would need to exceed -log10(0.05/100) = 3.30.

## Discussion

In spite of exhaustive efforts, it did not prove possible to find any genes which demonstrated even suggestive evidence of association with the phenotype tested. The results were just as would be expected under the null hypothesis. The 100 genes which seemed to demonstrate the most evidence in favour of association in the first phase of analysis showed no evidence at all for association in the second phase.

It may be worth reiterating that this kind of approach has been effective in the same sample in identifying genes implicated in the common, physical illness phenotypes mentioned above. For example, using hypertension and implementing a fixed weighting scheme for variants, 42 genes achieved MLP > 3 in the first phase of analysis with 200,000 participants and went through to the second phase with 270,000 participants (Curtis, 2023a). *GUCY1A1*, which codes for a subunit of soluble guanylate cyclase, achieved an MLP of 5.54 in the first phase and 5.06 in the second, while *DBH*, which codes for the enzyme producing norepinephrine, produced an MLP of 3.4 in the first phase and 5.61 in the second (with variants in *DBH* reducing hypertension risk). Thus, both genes were clearly implicated at conventional levels of statistical significance, in sharp contrast to the results obtained from the current study.

An obvious limitation of the current study is that the phenotype is vague, poorly defined and perhaps overinclusive, given that it identifies around 10% of participants as cases. However, we can note that the hypertension phenotype was also rather weakly defined, consisting of anyone who either self-reported that they had high blood pressure or who was recorded as having a hypertension-related diagnosis or who was taking a medication commonly prescribed for hypertension (Curtis, 2023a). This algorithm resulted in over 35% of participants being classified as cases. Nevertheless, the methods used were still able to convincingly implicate biologically plausible genes. As previously stated, the phenotype chosen for this study was used because most participants had answered Yes or No to the question of whether they had seen a psychiatrist and because in the UK seeing a psychiatrist would indicate significant morbidity, as most mental disorder is dealt with in primary care. More detailed information regarding mental health is only available for a smaller proportion of participants. We also note that this phenotype has a high genetic correlation with the phenotype of MDD as used in the previous GWAS, suggesting that similar genetic variation contributes to both conditions.

Another team has carried out analyses of the same UK Biobank dataset using seven different definitions for depression (Tian *et al*., 2024). Although they were able to demonstrate that the overall genome-wide burden of LOF and rare damaging missense variants was associated with depression, no individual genes were statistically significant after correction for multiple testing. For two genes, *SLC2A1* and *NOG*, the evidence was reported as ‘suggestive’ but neither could be replicated in another dataset and neither seems biologically plausible. Considering that study alongside the present one, a variety of approaches to deriving a phenotype reflecting significant affective disorder have failed to implicate specific genes using this dataset.

The results obtained (or lack of them) suggest that relatively common mood disorders, even if severe enough to warrant referral to a specialist, are genetically too heterogeneous for rare variant analyses to be effective using realistic sample sizes. An alternative approach could be to use a much more restrictive phenotype definition aimed at focusing on very severe illness, such that the lifetime prevalence might be more in the region of 1%, comparable with schizophrenia. Such a phenotype might resemble that used for the CONVERGE genome-wide association study, defined as severe recurrent depression requiring hospital treatment and perhaps additionally restricted to include only cases of melancholia (Cai *et al*., 2015). The experience with schizophrenia and other non-Mendelian phenotypes suggests that numbers of cases running into the tens of thousands are required to implicate specific genes using rare variants identified through exome-sequencing studies (Backman *et al*., 2021; Singh & The Schizophrenia Exome Meta-Analysis (SCHEMA) Consortium, 2022; Wang *et al*., 2021). If the phenotype to be studied were depression so severe as to have a prevalence of only 1%, then a biobank sample drawn from the general population would need to have a total sample size of at least 2 million in order to expect that it might contain 20,000 cases. Alternatively, research subjects could be specifically recruited, which would still require a large, multicentre effort. While focussed recruitment might require a smaller total sample size, a disadvantage is that exome-sequencing might then allow detection of rare variant associations with depression but not with any other phenotypes. Whereas a biobank sample can provide information about a wide range of phenotypes, potentially adding value to the costs entailed in sequencing.

Whichever way one looks at it, the resource costs which would be involved in strategies providing a reasonable expectation of producing actionable results seem daunting. An alternative approach might be to temporarily abandon further attempts at elucidating depression genetics and instead to focus efforts on identifying genes impacting risk of bipolar disorder. The hope would then be that insights gained from bipolar disorder research into the biological mechanisms underlying control of affect might subsequently be applied to more focused attempts to elucidate the pathogenesis of depression. That said, at time of writing the only gene to be implicated in bipolar disorder risk, using exome sequence data from 14,000 cases, is *AKAP11* and its mechanism of action is far from clear (Palmer *et al*., 2022).

To conclude, this study utilising exome sequence data from over 50,000 cases with mood disorder sufficiently severe to warrant referral to a specialist fails to detect even a hint of a signal of association of rare, damaging variants within any specific gene.

## Supporting information

Curtis supplementary material 1 Curtis supplementary material

Curtis supplementary material 2 Curtis supplementary material

## Data Availability

The raw data is available on application to UK Biobank at https://ams.ukbiobank.ac.uk/ams/. Detailed results with variant counts cannot be made available because they might be used for subject identification. Software and scripts used to perform the analyses is available at https://github.com/davenomiddlenamecurtis.

## Supplementary material

The supplementary material for this article can be found at https://doi.org/10.1017/neu.2025.10025.
